# An Exploratory Study of a Choreographic Approach to Golf Swing Dynamics: Bridging Biomechanics and Laban Movement Analysis

**DOI:** 10.3390/s24216845

**Published:** 2024-10-24

**Authors:** Wangdo Kim, Albert H. Vette, Wanda Ottes, Colleen Wahl

**Affiliations:** 1 Ingenieria Mecanica, Universidad de Ingenieria y Tecnologia—UTEC, Lima 15063, Peru; 2 Department of Mechanical Engineering, University of Alberta, Edmonton, AB T6G 1H9, Canada; vette@ualberta.ca; 3 Department of Biomedical Engineering, University of Alberta, Edmonton, AB T6G 1H9, Canada; 4 Glenrose Rehabilitation Hospital, Edmonton, AB T5G 0B7, Canada; 5 Danstec, 9832PE Den Horn, The Netherlands; ottes015@planet.nl; 6 Integrated Movement Studies, Alfred University, Alfred, NY 14802, USA; wahl@alfred.edu

**Keywords:** Laban Movement Analysis, inertial tensor, biomechanical analysis, screw algebra, motion tracking sensors, neurorehabilitation

## Abstract

This study introduces an innovative integration of Laban Movement Analysis (LMA) with biomechanical principles to examine the golf swing dynamics from an ecological perspective. Traditionally, LMA focuses on the qualitative aspects of movement, often isolated from external influences. This research bridges that gap by investigating how golfers manage and adapt to the inertial forces of the club throughout the swing. Using motion tracking sensors and screw theory, we analyzed the spatial movement pattern in the Kinesphere (mapped as an icosahedron) and related it to force dynamics in the Effort Cube through the inertia tensor. The results showed significant differences between skilled and novice golfers in terms of how efficiently they align their movements with the club’s inertia. Skilled golfers demonstrated smoother Instantaneous Screw Axes (ISAs) and better synchronization with inertia forces, while novice golfers exhibited more abrupt deviations. These findings suggest that integrating qualitative movement descriptors with biomechanical models provides deeper insights into swing efficiency, performance improvement, and injury prevention. This combined framework offers a novel method to enhance both qualitative and quantitative analysis of golf swings.

## 1. Introduction

Golf is a sport that requires a complex interplay of physical, technical, and strategic skills. Among these, the biomechanical analysis of the golf swing stands out due to its critical role in performance enhancement and injury prevention. The golf swing is a dynamic movement that involves the entire body in a coordinated sequence, with the downswing phase being particularly crucial for the generation of speed and power. Traditionally, research in this area has focused on kinematic and kinetic aspects, such as body alignments, joint angles, and the forces exerted during the swing [1,2]. Researchers have reported on the kinematics and kinetics of the golf swing in terms of the relative contribution of each of the anatomical structures that are involved [3,4,5,6,7,8]. However, these mechanical descriptions often do not fully capture the qualitative aspects of movement fluidity and efficiency that distinguish skilled golfers.

The Laban/Bartenieff Movement System (LBMS) offers a comprehensive theoretical framework for understanding human movement through three aspects: Doing (embodying), Seeing (observing), and Writing (notating or motifing). This system, developed by Rudolf Laban and his colleagues, describes body movements in terms of Body, Effort, Space, and Shape (BESS) [9,10].

Effort, which Laban referred to as the dynamics of movement, involves understanding the dynamic intention and energy behind movements. The Effort category addresses the energetic expression of movement through four motion factors:

1. Weight (Pressure, Resistance): LIGHT–STRONG;
2. Time (Fitting to a timeframe): SUSTAINED–QUICK;
3. Space (Focus, Attention): INDIRECT–DIRECT;
4. Flow (Movement dynamic): FREE–BOUND.

Each of these factors has two polarities, allowing for a nuanced description of movement quality.

It is important to distinguish between two uses of the term “Space” in Laban Movement Analysis:

1. Space as an Effort factor: This refers to the quality of attention or focus in movement, which can be direct or indirect.
2. Space as a general category: This relates to the structure of space around the mover, including concepts such as Kinesphere, Platonic solids, points, and pathways.

**Figure 1:**
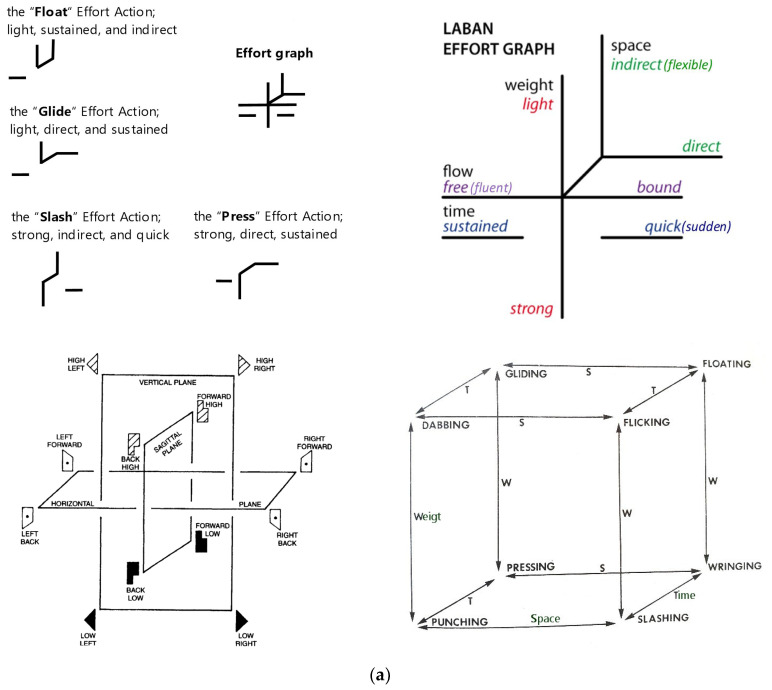

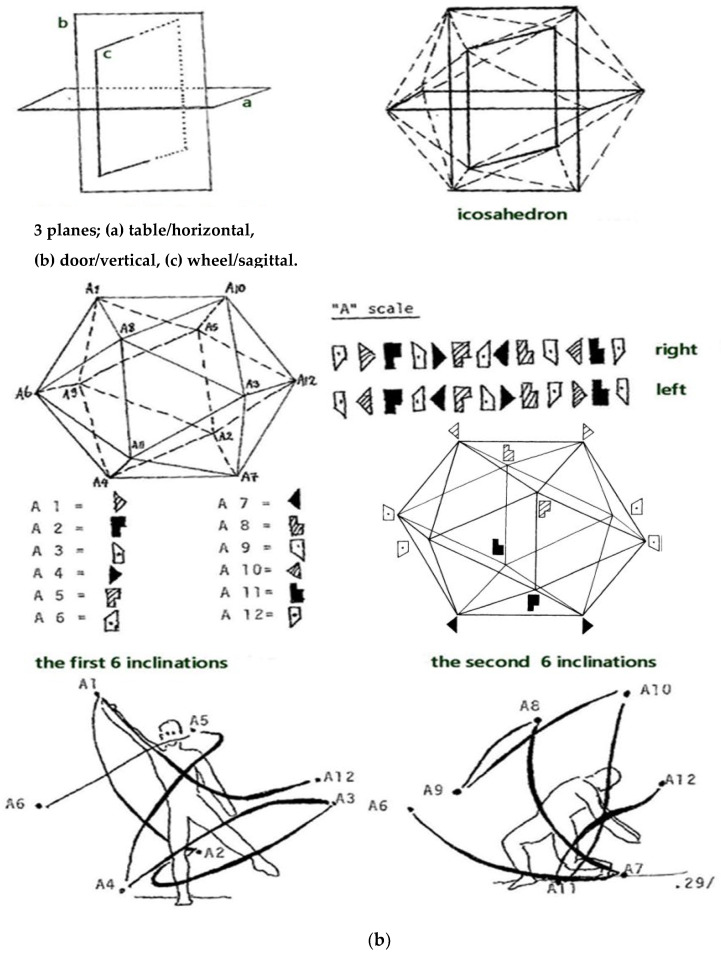
(**a**) Laban’s Effort Graph. This Effort graph, created by Rudolf Laban, illustrates the organization of inner intent or motivation behind a movement. The graph delineates the polarities of the four Effort qualities: Weight, Time, Space, and Flow. Each Effort quality has two opposing characteristics: Weight Effort ranges from light to strong, indicating the force exerted in a movement. Time Effort ranges from sustained to quick, reflecting the speed and acceleration of a movement. Space Effort ranges from direct to indirect, representing the focus and clarity of the movement’s path. Flow Effort ranges from free to bound, describing the continuity and control of the movement. The bottom-left panels show true 3D directions, illustrating the spatial orientation of movements, while the bottom-right panel shows the “Effort cube”, representing the combination of Effort elements in a three-dimensional framework. Effort quality in the context of the swing also directly impacts efficiency and timing. For instance, a ‘free flow’ in the effort action ‘press’—strong, direct, and sustained—can be seen in the even application of force through the impact with the ball, maximizing transfer of energy without unnecessary resistance. The implementation of LMA in this context serves as an innovative method to convey the complex biomechanical and qualitative nuances of the golf swing. The inclusion of these Effort Actions within training regimens may enhance a golfer’s understanding of the physical and psychological elements at play, potentially leading to improved performance and a deeper appreciation for the subtleties of the sport. (**b**) The ‘A’ scale inclinations illustrate the specific body alignments and movements associated with various phases of the golf swing. These inclinations represent the movements across the three spatial planes—horizontal, vertical, and sagittal. The scale integrates these planes through a series of 12 transversal units, comprising six on the right and six on the left, capturing a range of motions from flat and steep to flowing. This structure effectively links the angular dimensions of the three planes to the dynamic movements of the golfer.

For example, when discussing the Effort Cube (which is actually a cube as shown in Figure 1a), we are referring to the spatial orientation of different Effort combinations. On the other hand, when talking about space in terms of Kinesphere or scales (as described in Figure 1b), we are addressing the broader spatial concepts of Laban Movement Analysis. This distinction ensures clarity in our discussion of movement dynamics and spatial concepts within the LBMS framework.

Despite the comprehensive nature of biomechanical models, there remains a research gap in how these models integrate qualitative movement descriptors that can differentiate between skill levels and identify movement efficiency. The application of LMA, particularly its Effort component, offers a promising avenue to fill this gap. By analyzing golf swings through the lens of Effort qualities such as the free or bound nature of flow, researchers can explore how these dynamics correlate with traditional biomechanical measurements, such as the Instantaneous Screw Axis (ISA), which describes the rotation and translation of body segments [11]. One form of representing the club’s motion is the Instantaneous Screw Axis (ISA) [12,13].

Despite the potential applicability of LMA in sports sciences, very few studies, if any, have utilized this rich analytical framework to understand and enhance the biomechanics of golf swings or other sports activities. This represents a significant research gap, as LMA can provide novel insights into the qualitative aspects that make an efficient or powerful golf swing. Notably, the incorporation of LMA into sports biomechanics remains largely unexplored, marking this study as a pioneering effort to integrate these qualitative descriptors with quantitative biomechanical models.

The aim of this study is to investigate how golfers manage the inertia of the club and how these dynamics differentiate skilled golfers from novices. The primary independent variables in this study are as follows:

Instantaneous Screw Axis (ISA): A key biomechanical descriptor representing the axis around which both rotation and translation occur during the swing. This variable provides insights into the efficiency and synchronization of the golfer’s movements with the club.

Principal Axes of Inertia: The directions along which the club’s mass is distributed, influencing how the golfer must adjust their movement to handle the changing forces throughout the swing.

By analyzing these variables, we seek to uncover how golfers adapt their movements to the evolving inertia of the club during different phases of the swing. The interaction between the ISA and the principal axes of inertia is central to understanding how movement efficiency is achieved and how it varies between different skill levels.

Through this dual framework, combining ISA and inertia-based analysis with LMA’s qualitative descriptors, this study provides a more holistic understanding of golf swing dynamics. These findings will offer actionable insights for enhancing golf performance and reducing the risk of injury by addressing both the mechanical and qualitative aspects of movement.

## 2. Materials and Methods

### 2.1. Instrumentation and Data Analysis

In this study, rather than collecting new empirical data, we utilized an existing dataset from previous research that detailed the golf swings of two female golfers with different skill levels [14] (Table 1). This dataset was chosen for its comprehensive capture of biomechanical movements using high-precision motion capture technology and ground reaction force measurements, making it highly suitable for our analysis.

**Figure 2:**
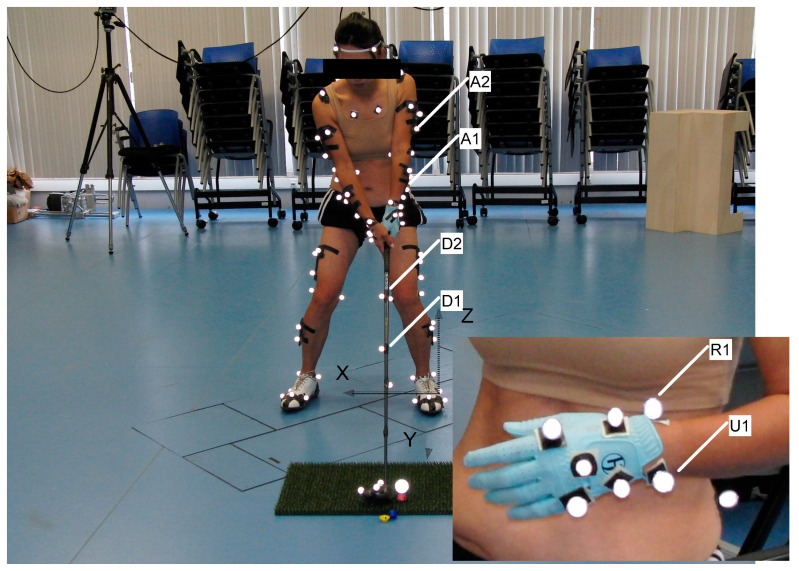
Displayed here are the positions of reflective markers on a golfer at the moment the club addresses the ball. This setup was used to capture the biomechanical data originally recorded, detailing the anatomical landmarks critical for analyzing movement dynamics. The separate panel shows detailed placements on the hand, essential for understanding the grip dynamics and the resultant force transmission through the golfer’s body during the swing. The origin of the global frame coincides with the first COP location of the left foot.

The original data were collected using a sophisticated array of twelve high-speed Qualisys cameras, which accurately recorded the three-dimensional positions of reflective markers attached to the golfers and their clubs. This setup was designed according to the standards of the International Society for the Advancement of Kinanthropometry, ensuring that the biomechanical analysis adhered to high measurement accuracy and reliability (Figure 2). Ground reaction forces were measured using a Kistler force plate, providing essential data on the biomechanical forces exerted during the swings.

By reusing this existing dataset, we were able to directly apply our analytical models without the variability and resource constraints associated with new data collection. This approach also allowed us to focus on in-depth analysis using established data, ensuring that our study was both resource efficient and grounded in reliable biomechanical metrics. The dataset included detailed annotations of each golfer’s movements and the corresponding biomechanical outputs, which facilitated a nuanced analysis of the swing mechanics. A total of twenty-four reflective markers and four marker clusters were used to reconstruct twelve body segments, including the head, torso, upper arms, forearms, hands, pelvis, thighs, shanks, and feet, providing a detailed representation of the golfer’s body dynamics during the swing for a comprehensive biomechanical analysis. Additionally, specific markers were placed on the club and wrist joint to define their respective reference frames. Marker coordinates for the period between the beginning of the downswing of a golf swing and up to the instant before impact were then acquired.

Motion capture was undertaken using an optoelectronic system of twelve Qualisys cameras (type: Oqus-300, Qualisys AB, Göteborg, Sweden) operating at 300 Hz. The collected data were processed using Qualisys Track Manager (QTM) software version 2.6 to ensure accurate tracking and reconstruction of the marker trajectories. For the club, markers were placed at specific points along the shaft and clubhead to capture its motion accurately. The wrist joint was defined using markers placed on anatomical landmarks around the wrist to establish a local reference frame.

Using the predefined anatomical coordinate system from the original study, which was based on key landmarks identified on the golfer and the club, we analyzed the motion data to extract the Instantaneous Screw Axes (ISAs) and their evolution during the downswing phase. This previously recorded data provided a robust basis for exploring how biomechanical properties such as the ISAs correlate with golfer skill level and technique efficacy. Reusing data from a well-designed previous study can enhance the efficiency of the research process and contribute to the sustainability of research practices by utilizing existing resources. This method helps to ensure that findings are based on previously validated data, which can support the reliability of the conclusions drawn from the analysis.

Force Space Mapping via the Inertia Tensor: To connect the kinematic data with the force dynamics, we applied the inertia tensor to map the golfers’ spatial acceleration space in the Kinesphere (represented as an icosahedron) to the force space (Effort Cube). This mapping allowed us to analyze how golfers manage and adapt to the inertial forces of the club during different swing phases. The inertia tensor serves as a mathematical representation of how the mass and shape of the club influence the forces experienced by the golfer.

Inertia Tensor Calculation: The inertia tensor was computed for each swing, taking into account the mass, center of mass, and geometric distribution of the club relative to the wrist joint. This tensor was then decomposed into principal axes of inertia, allowing us to quantify how the club’s inertia influenced the golfer’s movements.

Principal Axes of Inertia: By comparing the principal axes of inertia to the calculated ISA, we were able to determine how well the golfer’s movements aligned with the club’s inertia. Skilled golfers were able to maintain alignment with these axes, resulting in more efficient and powerful swings, whereas novice golfers struggled to maintain this alignment.

Screw Theory and Volute Phrasing: The biomechanical analysis was further enhanced through the application of screw theory, which provides a unified framework for describing both the rotational and translational aspects of the golfer’s movement. This allowed us to quantify the three-dimensional spatial pulls that occur during the swing.

Volute Phrasing: Laban’s concept of volute phrasing, which describes three unequal spatial pulls that change in a graded, proportional manner, was applied to analyze how the golfers adapted to the club’s inertia. This phrasing allowed us to identify how golfers transitioned between phases of the swing (e.g., from backswing to downswing) and how they managed the changing forces.

### 2.2. A Brief Overview of the Framework of Laban Movement Analysis

Laban’s Eight Effort Actions, also known as Laban Effort Drives, are a key part of Rudolf Laban’s system for understanding and notating dance and movement, known as Laban Movement Analysis (LMA) [15,16]. These Effort Actions are used to describe the quality of movement and are organized according to Laban’s Effort-Shape theory, which considers both the inner intention and the outer shape of the movement. The eight Effort Actions are categorized into four pairs of opposite qualities, with each pair representing one of the factors of motion: Weight, Time, Space, and Flow (Figure 1a).

The golf swing, a sophisticated biomechanical action, encapsulates these Effort elements in its execution. The swing begins with a preparatory phase where the golfer aligns with the target, transitioning to the backswing, culminating in the powerful downswing. Each phase can be mapped to Laban’s Effort Actions, offering a qualitative lens through which to analyze and refine movement (Figure 1b). The icosahedrons in Figure 1b are depicted from different perspectives to provide a comprehensive understanding of spatial orientations. The icosahedron on the left-side is viewed from the front, showing “A4” on the left and “A7” on the right. Conversely, the icosahedron below and to the right is viewed from behind the subject, with “A4” on the right and “A7” on the left. This distinction helps illustrate the spatial dynamics and orientations from multiple viewpoints, enhancing the analysis of movement.

Laban’s space harmony offers a unique perspective on the golf swing through the lens of the icosahedron and the A-scale: The A-scale, as shown in Figure 1b, is a specific movement sequence that demonstrates the first half and second half of the A-scale, right arm leading, with volute phrasing [17]. The A-scale, originally designed for analyzing fencing movements, can be applied to golf to understand the swing better. Here is a simplified explanation:

1. A-scale Structure:
  ○ The A-scale consists of 12 movement inclinations.
  ○ These are divided into two sets of 6 movements each.
  ○ The second set of 6 movements mirrors or parallels the first set.
2. Volute Phrasing:
  ○ “Volute” refers to a spiral or scroll-like form.
  ○ In movement terms, it implies a cyclical, flowing sequence that returns to its starting point.
  ○ Each volute in the A-scale comprises 6 movements.
3. Application to Golf Swing:
  ○ The first volute (6 movements) corresponds to the backswing and the start of the downswing.
  ○ The second volute (next 6 movements) represents the completion of the downswing and follow-through.
4. Movement Progression:
  ○ Each inclination in the scale represents a specific direction and level in space.
  ○ The movements flow from one to another in a predetermined sequence.
  ○ This sequence ensures a harmonious transition between different spatial pulls.
5. Body Coordination:
  ○ In the context of golf, the first inclination of each volute might involve a parallel leg movement on the active side of the body.
  ○ The second inclination could be represented by a step of the same leg in the direction of the final goal (towards the target in golf).
6. Fluidity and Symmetry:
  ○ The A-scale promotes smooth, flowing movement as arm and leg on the same side follow parallel trace forms.
  ○ While we focus on the right A-scale for a right-handed golf swing, there is a symmetrical left A-scale that mirrors these movements.
7. Spatial Harmony in Golf:
  ○ The A-scale helps illustrate how a golf swing is not just a back-and-forth motion, but a complex, three-dimensional movement pattern.
  ○ It shows how different parts of the body coordinate in space to create an efficient and powerful swing.
8. Analytical Tool:
  ○ By mapping parts of the golf swing to the A-scale, coaches and players can analyze the spatial efficiency and coordination of the swing.
  ○ It can help identify areas where the swing might deviate from an ideal spatial pattern.

Remember, the A-scale does not represent the entire golf swing, but rather provides a framework for understanding and potentially improving the spatial aspects of the swing. It is a tool that can offer insights into the complex spatial relationships involved in this athletic movement.

The bottom-left panel of Figure 1a illustrates true 3D directions, which are conceptually linked to the icosahedron depicted in Figure 1b. The icosahedron represents spatial orientations and movements, known as “Ai”, that are critical in understanding the spatial dynamics within Laban Movement Analysis. This linkage helps in visualizing how movements transition through different spatial planes and directions.

It is not compulsory to always use motions with 6 + 6 “Ai” or to include all the “Ai” in every analysis. However, incorporating these motions can be a very useful tool for golfers to become more aware of their bodies and their movements through space in all directions. These motions, known as choreutic forms or forms of space harmony, represent shapes and designs of energy around and through the space of the mover. They are oriented on a grid of the 27 directions, divided into three groups: the 6 simple high, wide up and down depth directions; right and left; and forwards and backwards. Additionally, there are the 8 high and lower corner diagonals and the 12 in-between directions on the horizontal, frontal, and sagittal planes, such as deep right, forward high, and center.

The 6 + 6 motions are particularly useful as they combine on-balance stability in a natural way, while simultaneously mobilizing forces of tilting, twisting, and the shifting of weight off the vertical line. By practicing these motions, golfers can improve their spatial awareness, balance, and coordination, which are essential for executing effective and efficient swings.

**Figure 3:**
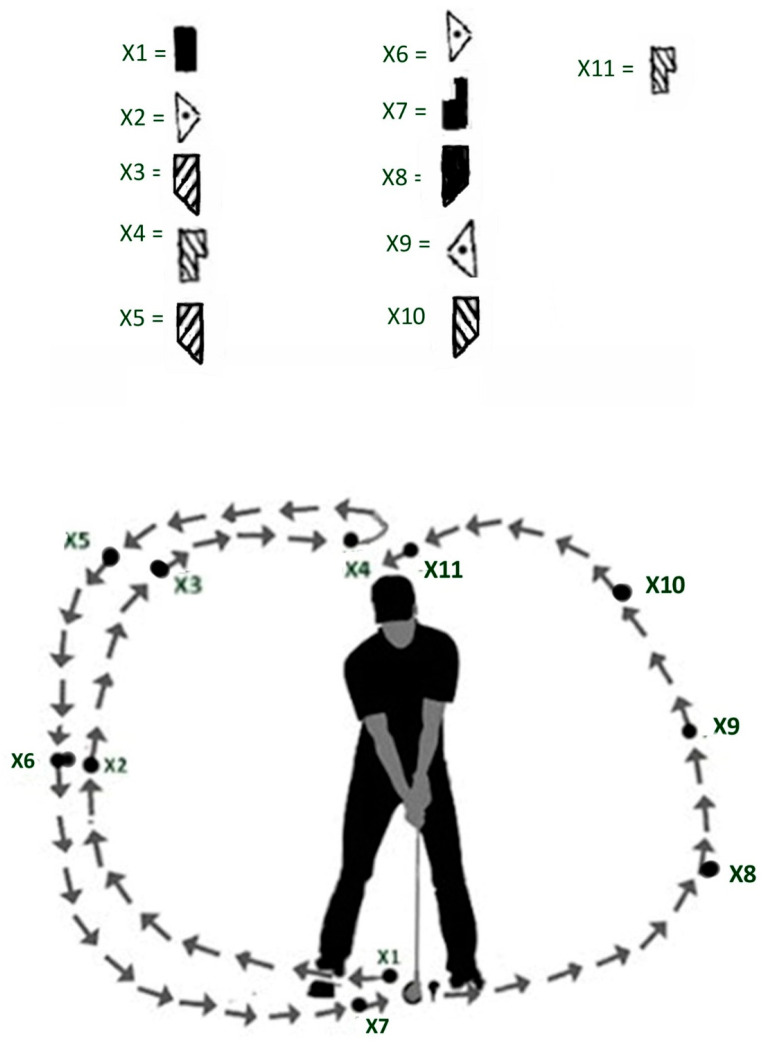
X-Scale positions in a golf swing. This figure illustrates the X-Scale positions specific to a golf swing, mapping key phases of the swing to spatial orientations and movements. Top Diagram: X1 to X11: Symbols representing key positions in the golf swing, indicating directional movements and spatial orientation in three planes (vertical, horizontal, and sagittal). Bottom Diagram: Golfer’s Swing Path: Arrows show the path of the golf club throughout the swing, from the start position to the follow-through, illustrating the continuous motion and flow. X1: Start position, club down in front of the golfer. X2: Right sideward middle (door plane, vertical). X3: Right backward high (table plane, horizontal). X4: Top of backswing, backward high (wheel plane, sagittal). X5: Right backward high (table plane, horizontal). X6: Right sideward middle (door plane, vertical). X7: Forward low (wheel plane, sagittal). X8: Left backward low (table plane, horizontal). X9: Left middle (door plane, vertical). X10: Left backward high (table plane, horizontal). X11: Follow-through, backward high (wheel plane, sagittal).

Building on this foundation, we have designated the term “X-scale” to describe a series of key positions throughout the golf swing, as shown in Figure 3. This designation is not a standard term in Laban Movement Analysis (LMA) but rather a label chosen for clarity and convenience within the context of this research. The X-scale is intended to illustrate the spatial and directional movements that a golfer performs during a swing, providing a framework for analyzing the golfer’s body mechanics and spatial orientation. By practicing and internalizing these movements, golfers can develop a heightened sense of spatial awareness and a smoother, more efficient swing.

Detailed Description of X-Scale Positions:

X1—Start Position (Downward in Front of Figure 3): The golfer’s initial stance with the club positioned downward in front of the body. This position sets the foundation for balance and alignment.

X2—Right Sideward Middle (Door Plane, Vertical): The club moves to the right side, halfway up in the vertical plane. This marks the early phase of the backswing, initiating the rotational movement.

X3—Right Backward High (Table Plane, Horizontal): The club continues upward and backward in the horizontal plane, reaching a high point. This position represents the mid-backswing, where the shoulders and hips rotate, and the weight shifts to the right leg.

X4—Backward High (Wheel Plane, Sagittal): At the top of the backswing, the club is positioned high and directly behind the golfer. This is a critical point where the potential energy is maximized, corresponding to A5 in the A-scale.

X5—Right Backward High (Table Plane, Horizontal): Similar to X3, this position reinforces the horizontal plane’s movement, emphasizing the rightward and backward trajectory during the backswing.

X6—Right Sideward Middle (Door Plane, Vertical): As the downswing begins, the club returns through the right side in the vertical plane. This transition involves uncoiling the body and shifting weight toward the left leg.

X7—Forward Low (Wheel Plane, Sagittal): The club moves forward and low, approaching the impact point. This position highlights the acceleration phase of the downswing, where speed and precision are crucial.

X8—Left Backward Low (Table Plane, Horizontal): Post-impact, the club follows through to the left and backward in the horizontal plane. This position indicates the initial phase of follow-through, where the golfer’s weight shifts fully to the left leg.

X9—Left Middle (Door Plane, Vertical): The club continues its path through the left side in the vertical plane. This phase ensures the continuation of the follow-through with maintained balance and coordination.

X10—Left Backward High (Table Plane, Horizontal): The club reaches a high point on the left side in the horizontal plane. This position represents the completion of the follow-through, with full body rotation.

X11—Backward High (Wheel Plane, Sagittal): The final position, where the club is high and behind the golfer, similar to X4. This position signifies the end of the swing, where the golfer’s body is fully extended and balanced, corresponding to A5 in the A-scale.

The X-scale is a practical application of the LMA principles, particularly the A-scale, in the context of golf. The A-scale provides a foundation for understanding movements in three directions, helping golfers develop a better spatial and body awareness. By practicing the X-scale, golfers can internalize the flow of movement, leading to a more natural and efficient swing.

The Effort Actions described in Table 2 for each phase of the golf swing can be mapped onto Laban’s Effort Graph (Figure 1a), providing a visual representation of the dynamic qualities of movement throughout the swing. This mapping allows us to see how the float, glide, slash, and free flow actions correspond to specific combinations of Effort qualities illustrated in the graph.

**Figure 4:**
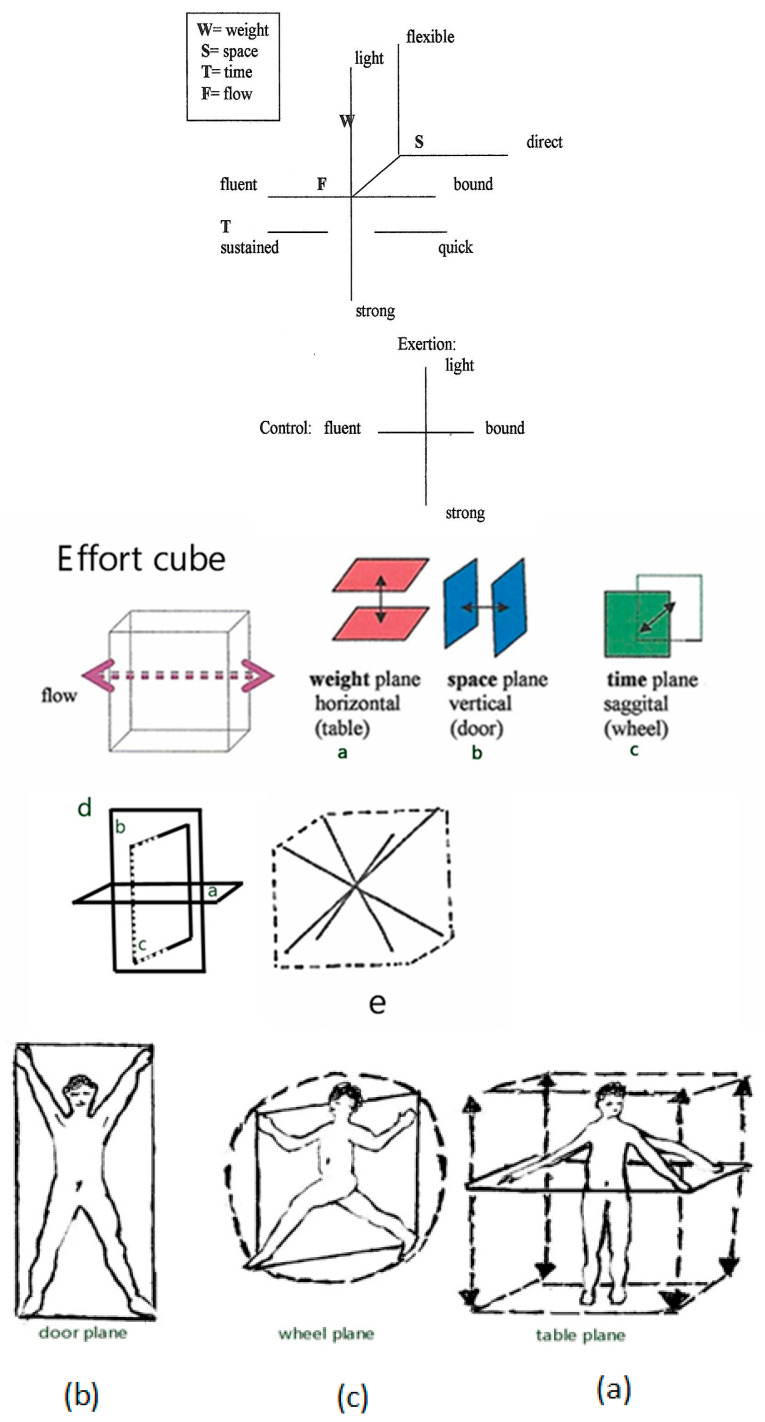
Comparison of Effort Cube panels. The figure demonstrates the integration of a, b, and c planes with arrows indicating the correct spatial orientations. The human figures expressing the three planes are labeled with the same a, b, and c labels for clarity. The red, blue, and green colors represent a two-dimensional projection of the Effort Cube, visualizing the “three unequal spatial pulls.” It is important to focus on the arrows to correctly interpret the spatial relationships depicted in the figure. This figure illustrates Laban’s Effort Graph diagram, which integrates three key dimensions of movement: (**a**) high/deep or the Weight plane, representing vertical movements and the distribution of weight; (**b**) side-to-side or the Space plane, depicting lateral movements and spatial orientation; (**c**) forward/backward or the Time plane, indicating movements related to timing and progression. (**d**) Shows a two-dimensional projection of the Effort Cube, simplifying the analysis by focusing on movement within a single plane. (**e**) Presents an X-marked schematic within the bounds of the Effort Cube, highlighting specific movement patterns and the intersections of the three dimensions. This figure is crucial for understanding how the dynamic qualities of movement are analyzed and interpreted using Laban’s framework.

The correct understanding of Figure 4 requires focusing on the arrows rather than the planes themselves. To aid in this interpretation, we will label the human figures expressing the three planes with the same a, b, and c labels. The colored red, blue, and green elements in the figure represent a two-dimensional projection of the Effort Cube, visualizing the “three unequal spatial pulls” throughout the swing. We distinguish the Effort Cube, representing the qualitative dynamics of Weight, Time, Space, and Flow, from the true 3D motions depicted by the icosahedron/Ai inclinations. This distinction clarifies how each analytical tool contributes to understanding the golf swing’s spatial and dynamic aspects.

The X-Scale provides a structured approach to analyzing the spatial and directional aspects of the golf swing, aiding in the development of spatial awareness and flow in the golfer’s movement.

### 2.3. Integrating Laban Movement Analysis with Golf Swing Dynamics: Mapping from the Effort Cube to the Kinesphere Through the Inertia Tensor

Laban’s theory of movement, rooted in the spatial dynamics of the human body, suggests that individuals can extend into and manipulate their surrounding space without changing their location, which we shall call the “stance” [17]. This theoretical framework becomes particularly relevant in analyzing how a golfer modulates their movements in response to the golf club’s inertia during various swing phases. While Section 2.2 provided an overview of spatial interactions, here we focus on how shaping influences these interactions by accommodating the characteristics of the golf club’s inertia surface.

Figure 3 is instrumental in illustrating this concept. It shows not just the directional orientations and areas within the Kinesphere, but more importantly, how these are modulated through the golfer’s adaptive responses to the inertia properties of the golf club. The “Xi” symbols in Figure 3 transition from abstract indicators of Effort Actions to tangible demonstrations of how the golfer’s body shapes and conforms to the club’s inertia. The orientation of the 3D axes, clearly marked in the figure, helps delineate this process.

While Laban’s space harmonic research provided valuable insights into human movement, its application was limited by the use of Cartesian analysis, which does not fully capture the complex, three-dimensional nature of movement. To address this limitation and provide a more comprehensive understanding of the golf swing, we introduce screw theory as a unifying concept that combines rotation and translation into a single theoretical model.

The golf swing, particularly the downswing phase, exemplifies what Laban referred to as volute phrasing [17]. This involves three unequal spatial pulls that constantly change their relationship to each other through transverse movement. This movement can be likened to a three-dimensional spiral, where vertical, sagittal, and horizontal components change in a graded, proportionate way, cutting or sweeping through space.

This integration of screw theory with Laban’s concepts provides a powerful tool for understanding the complex spatial relationships and dynamic qualities of the golf swing, offering insights that can be applied to both skilled and novice golfers. It allows us to quantify and visualize the “three unequal spatial pulls” [17] and their constant changes throughout the swing, providing a more accurate representation of the movement’s space harmonic qualities.

This study employs screw theory [18,19] as a foundational mathematical framework to describe the spatial vector quantities relevant to the golf swing. Screw theory facilitates the representation of the six degrees of freedom of a rigid body (three translational and three rotational) in a unified manner. By defining the Instantaneous Screw Axis (ISA) of the golf club, we capture both the rotational and translational dynamics essential for analyzing the complex movements in golf.

**Figure 5:**
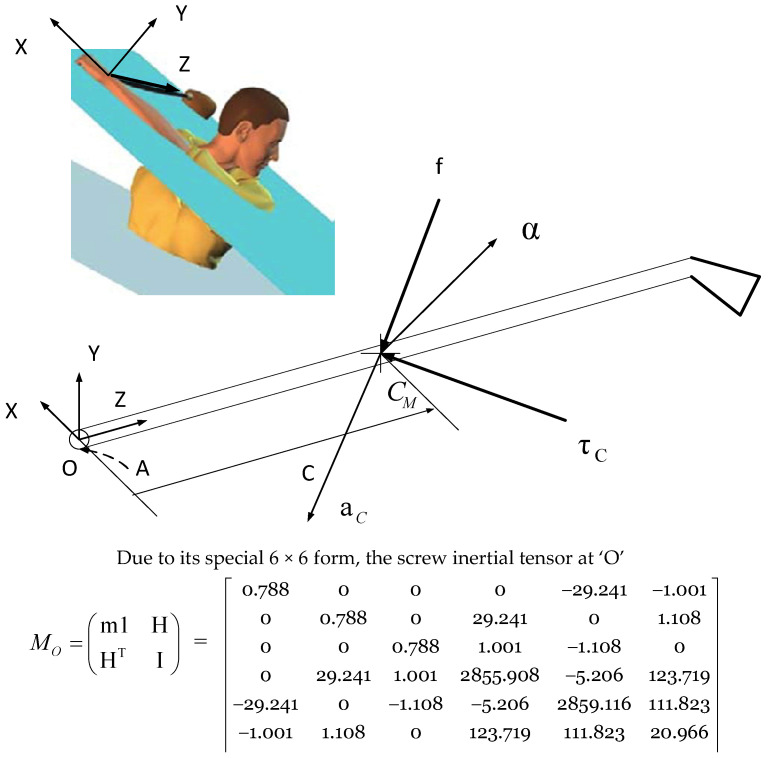
This figure illustrates the grip reference frame attached at the center of the end of the club shaft. The spatial inertia tensor, computed with reference to the origin of the grip coordinate system, is depicted showing its principal axes and moments of inertia. This representation highlights how the inertia tensor transforms at any point along the wrist joint axis ‘A’, emphasizing the consistent eigenvalues irrespective of the positioning. The spatial arrangement allows us to explore how the inertia impacts the golfer’s control over the club during dynamic movements.

Consider the situation in Figure 5: A club has a mass, *m*; its center of mass, *C_M_*, is given by the position vector, *C*; and the inertia tensor about its center of mass is *J*. The club is at rest, and experiences a force, *f*, acting along a line passing through the center of mass, and a couple, *τ_C_*. The resulting acceleration is determined by an angular acceleration, α, along an axis passing through the center of mass (*C_M_*), the linear acceleration, *a_C_*, on *C_M_*.

To clarify our approach, we did not directly measure the inertia tensor. Instead, we used data obtained from a club manufactured by the same company as the one used in our experiment. We then applied a geometric scaling method to these data to estimate the mass moment of inertia for our specific club. This scaling was based on the principle, as detailed by Zatsiorsky (1998) in Section 4.4.2 of his book [20], that the moments of inertia of each segment are proportional to the mass times the square of a linear dimension.

Furthermore, we fine-tuned the segment parameters (including mass, center of mass location, and inertia) using the method described by MacKenzie and Sprigings (2009) [4]. This tuning process allowed us to adjust the parameters to match the specific club used in our experiment. The resulting inertia tensor, presented in Figure 5, is therefore a product of this scaled and tuned approach, derived from established data and methodologies, rather than direct measurement. This method allowed us to obtain a reliable estimate of the inertial properties while accounting for the specific characteristics of our test club.

Therefore, the inertia matrix included in Figure 5 is a result of our own calculations based on these established methods and datasets (units in $\mathrm{kg}\cdot {\mathrm{cm}}^{2}$). As such, the matrix is our original work, derived from the referenced data.

The equation of motion is considered as a mapping from the twist-like screw acceleration to a wrench space [14].

(1) $$\left[\begin{array}{c} f\\ \tau_{c} \end{array}\right]=\left[\begin{array}{cc} m1 & 0\\ 0 & J \end{array}\right]\left[\begin{array}{c} a_{c}\\ \alpha \end{array}\right]$$

where $m1=\left[\begin{array}{ccc} m & & \\ & m & \\ & & m \end{array}\right]$== M, and 1 is the 3×3 identity matrix.

Since all of the spatial quantities are referred to with respect to the center of mass, the linear and angular components of motion are decoupled—the linear acceleration being entirely due to the force, and the angular acceleration being a result of the couple. To transform Equation (1) into the origin of the joint axis (Figure 5), we obtain the following:

(2) $$\left[\begin{array}{c} f\\ \tau_{0} \end{array}\right]=\left[\begin{array}{cc} m1 & H\\ H^{T} & I \end{array}\right]\left[\begin{array}{c} a_{0}\\ \alpha \end{array}\right]$$

where *H* = *C* × *M*, *I* = *J* + *C* × *MC* ×*^T^*, and *C*× is the anti-symmetric skew matrix corresponding to C.

(3) $$C\times =\left[\begin{array}{ccc} 0 & -C_{z} & C_{y}\\ C_{z} & 0 & {-C}_{x}\\ {-C}_{y} & C_{x} & 0 \end{array}\right]$$

Due to its special 6×6 form, the spatial inertial tensor is as follows:

(4) $$M_{0}=\left[\begin{array}{cc} m1 & H\\ H^{T} & I \end{array}\right]$$

is expected to have special eigen structures.

The spatial inertia tensor *M*_0_ represented at the origin is a symmetric, positive definite tensor and transforms to any point ‘A’ by the spatial Jacobian, *Φ*, according to the following:

(5) $$M_{A}=\left[\begin{array}{cc} 1 & 0\\ -r_{A/0}\times & 1 \end{array}\right]M_{0}{\left[\begin{array}{cc} 1 & 0\\ -r_{A/0}\times & 1 \end{array}\right]}^{T}$$

where $r_{A/0}=\vec{OA}$ and $\Phi ={\left[\begin{array}{cc} 1 & 0\\ -r_{A/0}\times & 1 \end{array}\right]}^{T}$.

The eigenvalue problem provides a unique decomposition of *M*_0_ as follows:

(6) $$\left[\begin{array}{cc} m1 & H\\ H^{T} & I \end{array}\right]=M_{0}=\left[\begin{array}{cc} f & 0\\ \tau & \gamma \end{array}\right]\left[\begin{array}{cc} m_{f} & 0\\ 0 & m_{\gamma } \end{array}\right]{\left[\begin{array}{cc} f & 0\\ \tau & \gamma \end{array}\right]}^{T}$$

where *m_f_* and *m_γ_* are representing the eigen values of mass and mass moment of inertia, respectively (following the common notational tactics for the principal axes of inertia, we use $e_{1},e_{2},\;e_{3}$ for the corresponding eigenvectors to *m_γ_* and, to the corresponding principal moment of inertia, $I_{1}\leq I_{2}\leq I_{3}$ for *γ*). We state that *I*_3_ is the largest principal moment of inertia, representing the mass distribution along the longitudinal axis of the golf club. The *e*_3_ eigenvector, known as the principal axis of inertia, is aligned perpendicular to the longitudinal axis and passes through the mass center of the golf club. This alignment contributes to the perceptual simplifications obtained by using it as the axis of reference and is critical for understanding the dynamics of the golf swing.

One might wonder whether the decomposition based on the solution for the free-vector eigenvalue problem would be different at another point A. We apply the transformation rule (Equation (5)) to the above decomposition (Equation (6)) as follows:

(7) $$=\left[\begin{array}{cc} 1 & 0\\ -r_{A/0}\times & 1 \end{array}\right]\left[\begin{array}{cc} f & 0\\ \tau_{0} & \gamma \end{array}\right]\left[\begin{array}{cc} m_{f} & 0\\ 0 & m_{\gamma } \end{array}\right]{\left[\begin{array}{cc} f & 0\\ \tau_{0} & \gamma \end{array}\right]}^{T}{\left[\begin{array}{cc} 1 & 0\\ -r_{A/0}\times & 1 \end{array}\right]}^{T}$$

(8) $$=\left[\begin{array}{cc} f & 0\\ \tau_{0}-r_{A/0}\times f & \gamma \end{array}\right]\left[\begin{array}{cc} m_{f} & 0\\ 0 & m_{\gamma } \end{array}\right]{\left[\begin{array}{cc} f & 0\\ \tau_{0}-r_{A/0}\times f & \gamma \end{array}\right]}^{T}$$

(9) $$=\left[\begin{array}{cc} f & 0\\ \tau_{A} & \gamma \end{array}\right]\left[\begin{array}{cc} m_{f} & 0\\ 0 & m_{\gamma } \end{array}\right]{\left[\begin{array}{cc} f & 0\\ \tau_{A} & \gamma \end{array}\right]}^{T}$$

which shows that *M_A_* is decomposed by the same eigenscrews in the same manner, just represented at B. That is, no matter where the problem is posed, the same eigenscrews form the basis of the decomposition.

## 3. Results

### 3.1. A ‘Free’ Flow Versus a ‘Bound’ Flow Based on Laban’s Effort Theory

Our analysis of the previously collected data revealed distinct differences in the golf swing mechanics between skilled and novice players, particularly in how they manage the physical forces during the downswing. One skilled golfer exhibited a profound synchronization between the Instantaneous Screw Axis (ISA) and the dynamic forces exerted by the golf club’s inertia. This synchronization suggests a voluntary and strategic compliance with the natural forces, allowing these forces to guide the swing without opposition.

**Figure 6:**
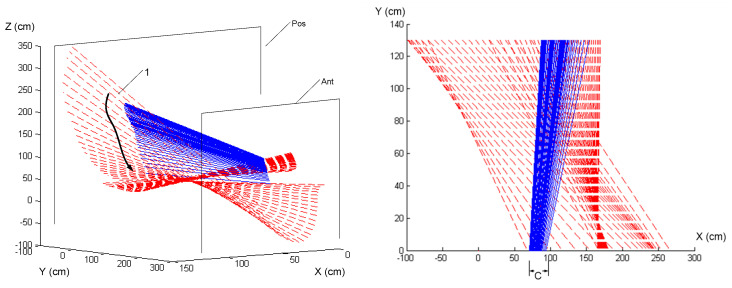
This figure illustrates the Instantaneous Screw Axis (ISA) represented for the golf club (solid lines depicted in blue) and the principal axes of inertia (e3 dashed lines depicted in red) during a novice golfer’s swing. The paths projected onto the medial and superior sides illustrate the motion dynamics, showing the lack of alignment between perceived inertia and actual movement paths, indicative of the novice’s struggle with effective force management and synchronization. The arrow indicates where the subsequent axes have migrated at every 0.0333 s of time step (units in cm). Adapted from [14], “Haptic Perception-Action Coupling Manifold of Effective Golf Swing”, International Journal of Golf Science, 2(1), 10–32. The axes reference their initial description and orientation as detailed in Figure 2.

**Figure 7:**
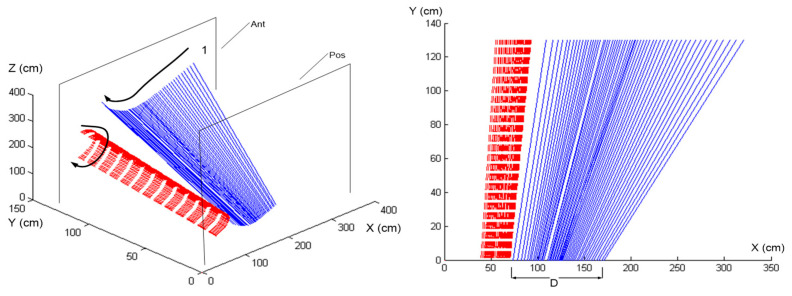
Showcased here is the proficient golfer’s ability to synchronize her swing dynamics effectively, as evidenced by the figure that illustrates alignment between the Instantaneous Screw Axis (ISA) represented for the golf club. and the e3 (dashed lines depicted in red). This alignment demonstrates her adept perception-action coupling, allowing her to voluntarily harness the club’s inertia to optimize swing mechanics and energy flow, contrasted with the novice’s disjointed motion paths. Adapted from [14] “Haptic Perception-Action Coupling Manifold of Effective Golf Swing”, International Journal of Golf Science, 2(1), 10–32.

Figure 6 and Figure 7 in this manuscript are adapted from our previous publication in the International Journal of Golf Science [14]. The original study, titled “Haptic Perception-Action Coupling Manifold of Effective Golf Swing”, provided foundational results that are further analyzed and expanded upon in this current research.

Figure 6 provides a dynamic visualization of the ISA paths during a novice golfer’s swing. The trajectories depicted by the solid lines and contrasted against the principal axes of inertia (dashed lines) illustrate a lack of alignment between perceived inertia and actual movement paths. This indicates a struggle with effective force management and synchronization, which is particularly evident from the disjointed ISA paths that exhibit abrupt changes in movement direction and speed. These perturbations suggest a resistance or a lack of coordination in allowing the inertia of the club to influence their body dynamics.

Conversely, Figure 7 illustrates the proficient golfer’s ability to synchronize her swing dynamics effectively. The alignment between the ISA (solid lines) and the e3 (dashed lines) demonstrates adept perception-action coupling, allowing her to voluntarily harness the club’s inertia to optimize swing mechanics and energy flow. This contrasted with the novice’s disjointed motion paths reveals significant insights into the qualitative differences in swing mechanics between different skill levels.

The movement patterns of skilled golfers demonstrated that their swings were not only synchronized in terms of timing but also optimized for energy utilization. The ISA paths in skilled golfers were smoother and showed a consistent alignment with the club’s inertia forces throughout the swing. This alignment allowed one skilled player to maintain an efficient kinetic chain that enhanced both the power and fluidity of the swing, indicative of her ability to harness and modulate the club’s inertia effectively.

In contrast, one novice golfer displayed multiple deviations from the ideal ISA path, with these deviations manifesting as abrupt changes in movement direction and speed. These perturbations indicate a resistance or a lack of coordination in allowing the inertia of the club to influence her body dynamics. The novice swings were characterized by a ‘Bound’ Flow, where the energy seemed restrained and movements were less fluid, likely resulting from a conscious or unconscious opposition to the inertia forces.

The spatiotemporal representation of the swings, especially the ISA paths, provided a vivid illustration of how the swing dynamics unfolded over time. For the skilled golfer, the ISA and the perceived inertia (e3 pathways) were closely aligned, suggesting a high degree of perceptual–motor integration. This integration enables proficient golfers to anticipate and adapt to the physical demands of the swing, allowing the natural progression of movement influenced by the club’s inertia. Conversely, the novice golfer’s e3 and ISA paths showed misalignment, indicating less effective perception-action coupling and a disjointed adaptation to the dynamic forces.

These results underscore the critical role of allowing physical forces to act naturally across the golfer’s biomechanical system, highlighting a fundamental difference in skill levels. The ability to voluntarily allow and adapt to these forces [16] is a hallmark of proficiency in golf, reflecting a deeper kinesthetic awareness and a more refined control over the complex dynamics of the golf swing.

### 3.2. Integrating Figure 3 and Figure 8 of the X-Scale

**Figure 8:**
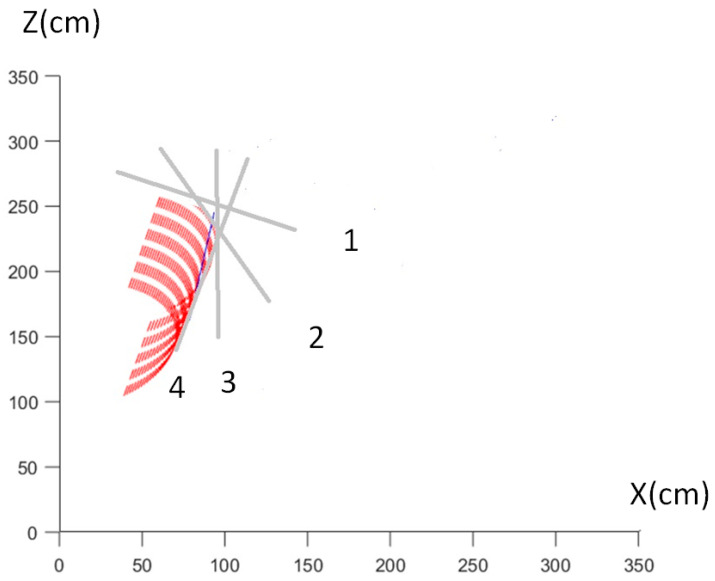
This image embodies the application of the LMA framework, illustrating how the golfer’s body dynamically adapts to the contour of the club’s inertia envelope during the downswing. Figure 8 is a projection of Figure 7 in the XZ plane, providing a detailed view of the spatial relationships and orientations of the vectors involved. The red dashed contour marks the principal axis of inertia of the club, serving as a guide that the golfer’s movements mold around. The e3 eigenvector, known as the principal axis of inertia, represents the largest principal moment of inertia and the mass distribution along the grey lines indeed represent the longitudinal axis of the golf club, aligned perpendicular to this axis and passing through the mass center of the golf club. The axes are defined as follows, consistent with Figure 2: X-axis is horizontal, Z-axis is vertical, and Y-axis is perpendicular to both the X and Z axes. This adaptive process, akin to a sculptor intuitively shaping clay, highlights the profound integration of body form with the evolving physical forces of the club, showcasing a sophisticated synchronization of movement and inertia for optimal swing efficiency.

Figure 8 encapsulates the concept of gathering/scattering as a shaping movement in golf, akin to Dell’s description of accommodating the plastic character of objects and molding space like a sculptor with clay [18]. The sequence from phases 1 to 4 demonstrates the golf club’s dynamic downswing, where the club’s motion is adapted and refined in response to the forces and trajectory required for an effective strike. Each line traces the club’s path, illustrating the adaptation of form and the creative interaction with space, capturing the expressive and sculptural qualities inherent in skilled sports movements.

Sequential visualization of a golf club’s downswing, highlighting the dynamic envelope of club inertia: Phases 1 to 4 illustrate the progressive motion of the club: starting from the initial downswing position (1), moving through the increasing acceleration (2, 3), and culminating in the final swing phase (4) where maximum velocity is achieved. The contour lines represent the club’s path, emphasizing how the inertia influences the trajectory and speed across the swing.

By integrating the positions from Figure 3 into Figure 8, we can illustrate how proficient golfers use the club’s inertia to guide the X-scale movements. This approach leverages the mapping strategies from the Effort Cube to the Kinesphere, allowing golfers to align their perceived virtual motion phases with the physical execution of the golf swing. Figure 8 is updated to include the following annotations and descriptions, demonstrating how each X-scale movement is influenced by the club’s inertia:

Label 1 (Top of Backswing):

X4: Top of backswing—backward high (wheel plane, sagittal):

At this point, the golfer utilizes the inertia of the club to achieve maximum extension and rotation, corresponding to A5 in the A-scale. This allows for efficient storage of potential energy.

Label 2 (Transition and Downswing):

X5: Transition—right backward high (table plane, horizontal):

The golfer transitions smoothly, using the club’s inertia to maintain balance and control.

X6: Early downswing—right sideward middle (door plane, vertical):

By harnessing the inertia, the golfer initiates the downswing with precision, ensuring the body aligns correctly.

X7: Forward low (wheel plane, sagittal):

The inertia aids in directing the club forward with controlled speed and accuracy, optimizing the kinetic energy transfer.

Label 3 (Impact):

X8: Left backward low (table plane, horizontal):

At impact, the golfer uses the club’s inertia to stabilize the motion, ensuring a solid hit.

X9: Left middle (door plane, vertical):

Post-impact, the inertia helps in maintaining the trajectory and balance.

X10: Left backward high (table plane, horizontal):

The golfer follows through with the club, leveraging inertia to complete the swing with fluidity.

Label 4 (Follow-Through):

X11: End of follow-through:

The final phase utilizes the remaining inertia to bring the club to a natural stop, ensuring the golfer remains balanced and ready for the next move.

By mapping these positions using the Effort Cube to Kinesphere strategies, proficient golfers can align their movements with the physical dynamics of the club, resulting in a more efficient and effective swing. This comprehensive approach not only enhances the golfer’s technical skills but also improves their overall performance by integrating spatial awareness and body mechanics seamlessly.

### 3.3. Motif Writing Using Laban Movement Analysis and Its Notation Are Tied Closely to the Depiction of the Golf Swing

**Figure 9:**
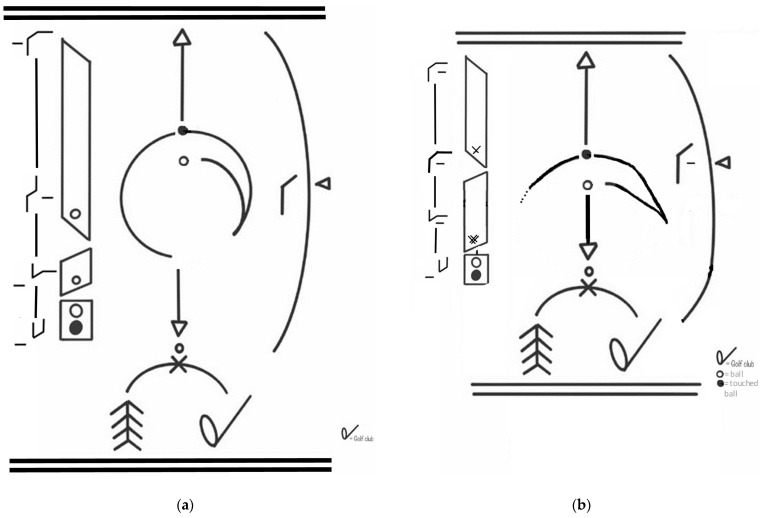
Illustration of the hands holding the drive and making a swing, along with additional information about the torso movements with the Effort elements. This figure uses Motif Writing to provide a simplified representation of key movement elements, rather than a complete notation of the entire swing. As a result, the weight shift is not visually represented in this figure. The central vertical line represents the body’s midline, with symbols on the left denoting body part positions and movements. The large curved arrow on the right depicts the swing arc. At the bottom center, a small circle with an “x” marks the golf ball’s position. Upward and downward arrows indicate the backswing and downswing movements. The sign at the bottom left represents the left hand and the right hand together in one symbol, indicating the coordinated use of both hands during the swing. Curved lines at the top and bottom frame the movement sequence. The legend distinguishes between the ball (open circle) and the touched ball (filled circle). This notation system translates the complex, three-dimensional golf swing into a detailed two-dimensional representation, encompassing spatial pathways, body engagement, and movement flow. The motif provides insights into the golfer’s technique, potentially revealing differences between novice (**b**) and proficient players (**a**) in terms of movement fluidity, body alignment, swing path consistency, weight shift, stability, and overall swing efficiency. This method, originating with Rudolf Laban, can become highly detailed. The accompanying sketches and notes provide context and clarity for those unfamiliar with the notation (Supplementary Materials).

Figure 9 presents the golf swing represented by applying Motif Writing (a simplified Labanotation system) and Effort Graph (notation form of quality of movement), which are both part of LMA. This figure serves to bridge the biomechanical analysis with the qualitative descriptions provided by LMA. Here, we explain the transition from the previous figures to Figure 9 and address its applicability to different skill levels of golfers.

Building upon the biomechanical foundation presented in Figure 6 and Figure 7, Figure 9 translates these parameters into the qualitative movement language of Labanotation. This system captures the essence of the golf swing by notating its spatial, temporal, and dynamic aspects. For a more comprehensive explanation of the Labanotation symbols and their interpretation in the context of the golf swing, please refer to Supplementary Materials. This system captures the essence of the movement by notating the spatial, temporal, and dynamic aspects of the golf swing. The Effort sign (Figure 9a) “Strong” is shown for the Effort Factor Weight, meaning “resolute with power” when returning from the backswing to impact (touching the ball). Vertical bows (Figure 9a) show that actions are performed simultaneously, show phrasing, and include body parts or add specific aspects to the movement. “Phrasing in movement” refers to how a sequence of movements is structured and connected, much like phrasing in music or language. It involves the flow and continuity of movements, highlighting the transitions and the relationship between different parts of the movement sequence. In the context of LMA and Labanotation, phrasing helps us to understand the dynamics, timing, and expressiveness of movement.

The depicted Labanotation score is not merely a static representation but a dynamic blueprint that outlines the essential phases of a golf swing. It visually encodes the golfer’s posture, the sequential flow of the swing, the directionality of the club, and the shifting weight and balance of the golfer’s body through space and time.

By applying the Labanotation to the golf swing, the analysis becomes a living, evolving process. The systems work both separately and in unison, providing tools to capture and enhance the expressivity and functionality of the golfer’s movements. They enable a deeper investigation into how the golfer’s body engages with and navigates through the biomechanical demands of the swing, aiming for efficiency, power, and harmony. Thus, Figure 9 does not merely depict the mechanical sequence of the golf swing but embodies a deeper dialogue between the golfer and the swing—a synthesis of kinetic elegance and biomechanical precision, continually evolving as our understanding of movement deepens. In conclusion, by incorporating Labanotation and Motif Writing into the analysis, Figure 9 provides a comprehensive visual representation of the golf swing’s dynamics and Effort qualities. This detailed explanation bridges the gap between previous figures and Figure 9, clarifying its application for different skill levels.

The Motif Writing analysis reveals distinct differences between novice and proficient golfers in their swing mechanics. Proficient golfers typically demonstrate smoother, more continuous lines in their movement flow (Figure 9a), particularly evident in the swing arc. Their body alignment symbols show greater consistency, and the swing path is more symmetrical and balanced. The weight shift is more pronounced and well timed (Figure 9a), with a more prominent grounding symbol indicating better stability. The follow-through of proficient golfers extends further, showing a complete and controlled motion. Overall, the spacing and arrangement of symbols in a proficient golfer’s motif suggest a more rhythmic and well-timed sequence of movements.

**Figure 10:**
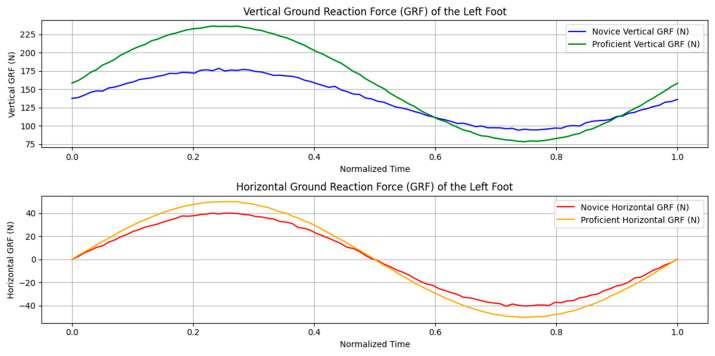
Ground Reaction Force (GRF) of the left foot during a golf swing for novice and proficient golfers. Top Panel: Vertical GRF for novice (blue) and proficient (green) golfers. The proficient golfer demonstrates higher and sharper peaks in the vertical GRF, indicating efficient force application during the downswing and impact phases. The novice golfer shows more variable and lower peak forces, reflecting less distinct timing and less efficient force application. Bottom Panel: Horizontal GRF for novice (red) and proficient (orange) golfers. The proficient golfer displays a smoother and more consistent force application with less noise, whereas the novice golfer exhibits more variability and less consistent force patterns, highlighting the differences in balance and coordination between the two skill levels. The normalized time axis represents the progression of the golf swing from start to finish, capturing the critical phases of the swing and their corresponding GRF profiles.

In contrast, novice golfers’ motifs often display more abrupt or segmented lines (Figure 9b), indicating less fluid transitions between swing phases. Their body alignment symbols may show greater variation, suggesting inconsistent positioning. The swing path for novices might exhibit deviations or asymmetries, while their weight shift appears less pronounced or poorly timed (Figure 10). The grounding symbol may be less defined, indicating less stable positioning throughout the swing. Novices’ motifs might also show additional symbols representing unnecessary movements or compensations, reflecting less efficient motion. These differences in the Laban motif visually capture the more refined, efficient, and controlled movements of proficient golfers compared to the less coordinated and consistent movements of novices. In Figure 9, the weight shift is not explicitly shown because this Labanotation primarily focuses on the hands holding the club and the swing motion, along with some information about torso movements and Effort elements. This is an example of Motif Writing, which is a simplified form of Labanotation that captures key aspects of the movement rather than providing a complete notation of the entire swing.

However, the concept of weight shift is better illustrated in Figure 4, which shows Laban’s Effort Cube. In this figure, the weight shift is represented in the vertical space plane of the Effort Cube. This plane demonstrates how the golfer’s weight moves up and down, as well as side to side, during the swing. The vertical space plane, moving through the door plane in the Kinesphere representation, coordinates the weight shaft dynamics effectively, reflecting the golfer’s ability to maintain balance and control throughout the swing.

## 4. Discussion

The current study integrates the principles of Laban Movement Analysis (LMA) and biomechanics to enhance the understanding and application of movement dynamics in golf swings. Specifically, it addresses the differentiation between the ‘A’ scale or ‘X’ scale, which represents the actual movement of a player, and the perceived virtual motion by the golfer to control the perception and action cycle.

### 4.1. Application of ‘X’ Scale to Actual Movement

The ‘X’ scale, as depicted in Figure 3, represents the true 3D directions and Effort Cube characteristics relevant to the golf swing. These scales are crucial in understanding the golfer’s physical movements. The positions X1 to X11 illustrate the precise biomechanical actions at different stages of the swing, highlighting how the golfer’s body moves through space. For instance, positions such as X4 (top of the backswing) and X8 (impact) show critical points where the golfer’s posture and force application are optimized for maximum efficiency and power. This approach provides a detailed biomechanical framework that can be used to analyze and improve a golfer’s physical performance through targeted training and adjustments.

By analyzing the Instantaneous Screw Axis (ISA) of the golfer’s twist of the club’s inertia, we observe harmonic ratios of effort in the three planes of flow. This harmonic swing tends to make the clubs commence to twist about the same ISA, which we refer to as Laban’s space harmonic. The ISA, as represented in Figure 6 and Figure 7, shows the complex motion patterns of the club, indicating how rotational and translational movements are synchronized during the swing to optimize performance and efficiency. Integrating these elements of LMA with biomechanical principles allows golfers to understand and improve critical aspects of their swing, enhancing overall performance and achieving a more efficient and powerful swing.

To bridge the theoretical concepts in this study with practical coaching methods, we propose the following strategies that coaches can use to incorporate Laban Movement Analysis (LMA) and biomechanical principles into golf training:

### 4.2. Synchronization of Body Movement with Club Inertia

Exercise Example: Coaches can introduce drills where golfers practice slow-motion swings while focusing on the sensation of the club’s weight and inertia. These exercises will help golfers become more aware of how their body naturally interacts with the club’s inertia, promoting smoother transitions between the backswing, downswing, and follow-through phases.

Coaching Tip: Instruct golfers to visualize the icosahedron (LMA’s Kinesphere) and mentally track their movement through space, paying attention to the feedback from the club’s inertia. By guiding the golfer’s attention to the relationship between body movement and club inertia, coaches can help improve synchronization and movement efficiency.

### 4.3. Application of Laban’s Effort Actions

Exercise Example: Coaches can design exercises that focus on specific Effort Actions such as float and slash. For instance, during the backswing, the golfer can practice a “floating” motion (light, sustained, indirect), followed by a more dynamic “slashing” motion (strong, quick, indirect) during the downswing. These motions correspond to the efficient transfer of energy from body to club.

Coaching Tip: Encourage golfers to explore different Effort combinations (e.g., glide for controlled movements, press for powerful movements) during practice sessions to refine control and fluidity. Coaches can use verbal cues and feedback to help golfers experiment with different energy levels in their swing, which may improve performance consistency and power delivery.

### 4.4. Improving Movement Fluidity

Exercise Example: A sequence of swings where golfers alternate between “free flow” (continuous, relaxed movement) and “bound flow” (controlled, restricted movement). This exercise helps golfers understand how excessive tension in their movements can lead to inefficiencies or injury, while also helping to enhance fluidity during the swing.

Coaching Tip: Coaches should emphasize minimizing unnecessary muscular tension during key phases of the swing (especially during the transition from backswing to downswing). This will allow golfers to transfer power more effectively without disrupting their kinetic chain.

## 5. Conclusions

The present study demonstrates the effective application of Laban Movement Analysis (LMA) and biomechanics in understanding and improving golf swing dynamics. By differentiating between the ‘X’ scale, representing actual movement, and virtual motion perception, we provide a holistic framework that encompasses both physical and cognitive aspects of the golfer’s performance.

Practical Implications: The findings highlight the importance of incorporating both biomechanical analysis and perceptual training in golf coaching. This approach can lead to more effective training regimens that address both the physical and mental aspects of the sport, ultimately improving performance and reducing the risk of injury.

Future Research: Further research is needed to explore the applications of this dual framework in other sports and activities. Additionally, advancements in VR and AR technologies could provide more immersive and accurate training environments, enhancing the effectiveness of perceptual and biomechanical training methods.

Scientific Contribution: This study bridges the gap between traditional biomechanical analysis and modern perceptual theories, offering a comprehensive model for understanding human movement in sports. By integrating these disciplines, we pave the way for innovative approaches in sports science and coaching.

In conclusion, the integration of Laban Movement Analysis with biomechanical principles offers a robust framework for analyzing and improving golf swing dynamics. This dual approach not only enhances technical performance but also aligns the golfer’s physical actions with their perceptual strategies, leading to a more efficient and enjoyable sporting experience.

## Figures and Tables

**Table 1:** This table provides demographic and golfing background information for the two female golfers who participated in this study. It includes age, height, mass, golf handicap, experience, and annual rounds played, offering context for understanding the differences in their biomechanical data and performance outcomes.

Participant	Age	Height	Mass	Handicap	Experience	Rounds
	(Years)	(cm)	(kg)		(Years)	(per Year)
A	17	167	54	32	1	10
B	51	165	55	8	15	110

**Table 2:** Mapping Laban Effort Actions to golf swing phases. This table illustrates the association between specific phases of the golf swing and the corresponding Laban Effort Actions (see Figure 1a for the signs of the four different phases). Each phase is characterized by distinct Effort qualities, providing a comprehensive understanding of the movement dynamics involved.

Golf Swing Phase	Laban Effort Action	Effort Qualities	Description
Preparatory Phase	Float	Light, Sustained, Indirect	Aligning body and mind with the target direction and outcome.
Backswing	Glide	Light, Direct, Sustained	Smoothly lifting the club, building potential energy.
Transition to Downswing	Slash	Strong, Indirect, Quick	Converting potential energy into kinetic energy.
Downswing	Free Flow	Free Flow	Fluid, coordinated movement to maximize impact efficiency.

## Data Availability

Data are contained within the article.

## Supplementary Materials

The following supporting information can be downloaded at: https://www.mdpi.com/article/10.3390/s24216845/s1, Figure S1: The basic forms of notation in Labanotation; Figure S2. The golf swing.
