# Supplementary material for: An Exploratory Study of a Choreographic Approach to Golf Swing Dynamics: Bridging Biomechanics and Laban Movement Analysis
Source: Sensors (Basel). 2024 Oct 24;24(21):6845. doi: 10.3390/s24216845 (PMC11548666; doi:10.3390/s24216845)
Supplement: Supplementary file 1 [file sensors-24-06845-s001.zip › sensors-3074064-supplementary.pdf]

Supplementary Materials

An annotated explanation of the symbols and motifs used in Figure 8, along with a detailed description of each component.

Figure S1. The basic forms of notation in Labanotation:

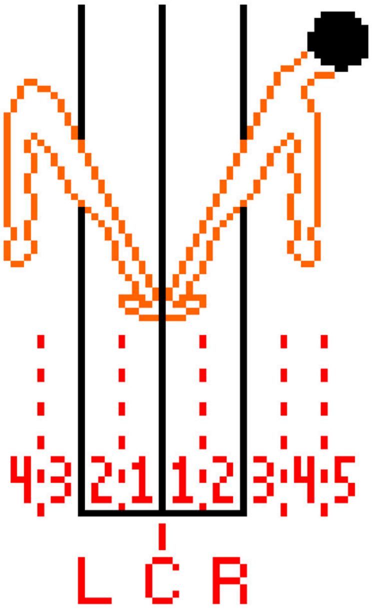

L = Left side
C = Centerline
R = Right side
1 = Support column
2 = Leg gesture column
3 = Body column
4 = Arm column
5 = Head column

The staff is read from the bottom to the top.

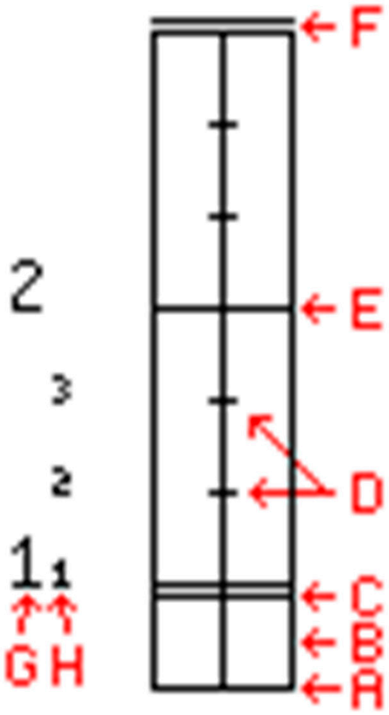

To structure the staff, different types of lines are used (similar to the bar lines in music notation). The individual beats in a bar always use a constant amount of space on the paper (in contrast to music notation where the space used on the paper is dependent on how much space is needed to write down the notes).

A = Line at the start of the staff
B = Starting position
C = Double line indicates the start of the movement
D = Short line for the beat
E = Bar line
F = Double line indicates the end of the movement
G = Large numbers for the bar
H = Small numbers for the beat (only in the first bar)

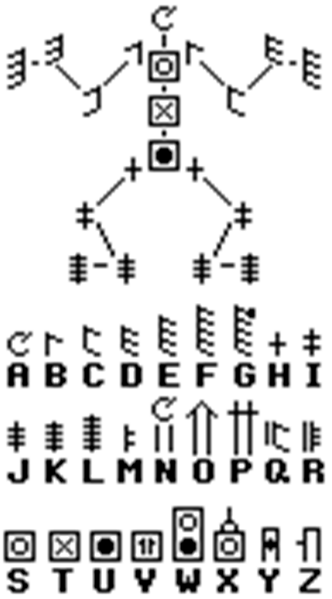

The body parts:

A = Head
B = Right shoulder
C = Right elbow
D = Wrist of right hand
E = Right hand F = Right fingers G = Right thumb
H = Hip I = Knee J = Ankle K = Foot L = Toes
M Right knee

Limbs:

In adding a double line to a join sign a limb sign is generated.
N = Neck O = Arm P = Leg
Q = Right upper arm
R = Right lower leg

Body areas:

By adding a box, a specific area of the body could be described.
S = Chest T = Waist U = Pelvis V = Shoulder section W = Whole torso

By adding pins, a specific surface of body areas could be described.
X = Upper front of chest Y = Palm or sole of foot Z = Thumb or big toe edge

The directions:

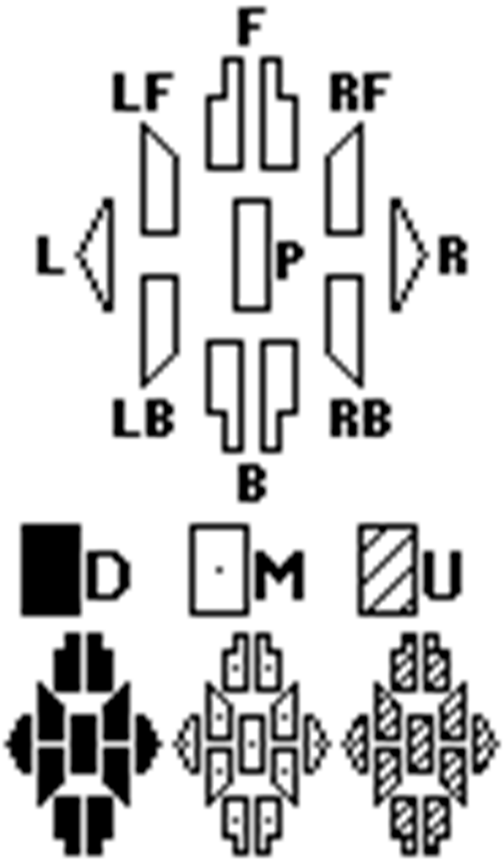

F = Forward
LF = Left forward
RF = Right forward
L = Left sidewards
P = At place
R = Right sidewards
LB = Left backwards
RB = Right backwards
B = Backwards

D = Downwards
M = In the middle
U = Upwards

Figure S2. The golf swing;

In Motif Writing, you can use the same symbols as in Labanotation, but in a simplified form.
But you still use a staff in the same way.

In Figure 9, the right-handed golf swing is notated where it shows the set-up with the golfclub in your hands and the golf ball in its place, the back swing, the return direction golf ball, the impact, and the finishing of the swing.
In addition, at the right, you see a longbow with the Effort sign "Strong" for the Effort Factor Weight, meaning "resolute with power" when returning from the backswing to impact (touching the ball).

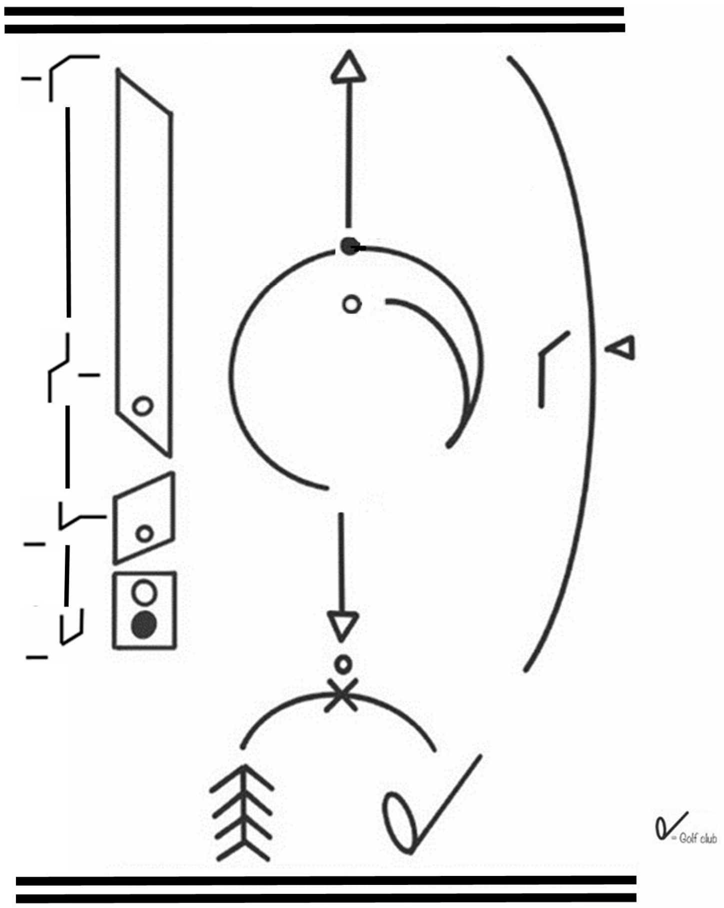

So we start with the double line indicating the start of the movement.

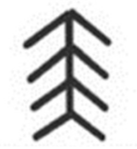 Next, you see the signs of the left and the right hand together in one symbol.

From the both hands sign to the golfclub sign (right), there is a bow with a single cross in the middle, and above the cross is the sign for the golf ball.

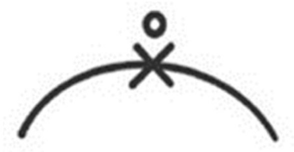

This bow indicates a connection meaning that the hands are holding the grip of the golf club (the bow) and that the golf club is addressed at the proper place of the ball.
By using space measurement signs, the narrow sign (the cross) shows a maximum of closeness (e.g. touching of the hand means grabbing).
The movement of the golf swing starts here.

Thereafter, we see the “design drawing”, a specific design to show what movement is given.
You can move in the following directions, with the next signs:

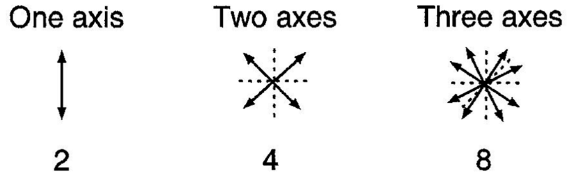

In three-dimensional space, you can choose different ways to choose those axes:

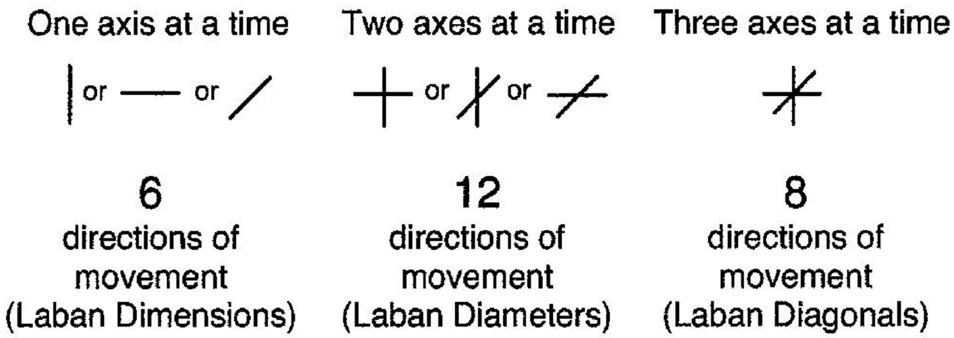

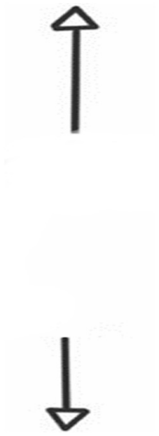

Here, it is the traveling path, the direction of movement dimension in one direction, “One Axis”.
The exact movement is drawn in the open space between the arrow lines up and down.

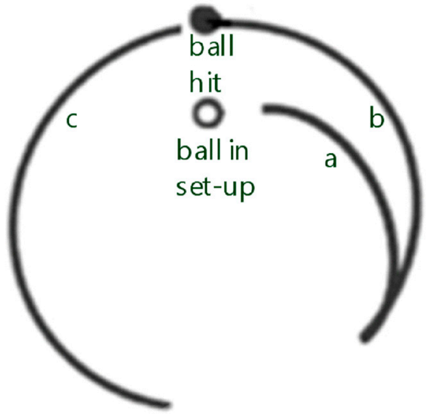

The movement drawn in the "design drawing" is in this case the twisted, spiral (screw) which is a basic shape form, an aerial movement. Here, the golf swing is made visible by the set-up with the golfclub in your hands and the golf ball on its place , the back swing (a), the return direction golf ball (b), the impact (the touching of the ball), and the finishing of the swing left back (c).
The ball has been touched (black) and flies away (not notated) in the direction of the pillow, straight forward.

In the score at the left side of the design drawing, we see the following signs;

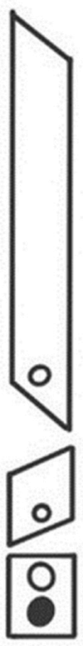

c

c) The **O** is the sign for the chest and is placed with the sign for a twist in the direction "counter clockwise".

b

b) The **o** is the sign for the chest and is placed in one of the signs of turns, here giving a twist in the direction "clockwise".

a

a) Belongs to the signs for body portions, which is shown here as the specified sign for the whole torso.

At the right side of the score there are 3 signs:

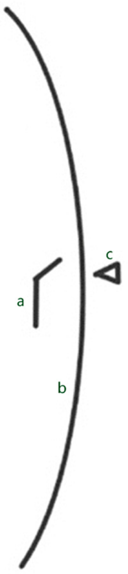

a)
The Effort sign "Strong" for the Effort Factor Weight means "resolute with power" when returning from the backswing to impact (touching the ball).

b)Vertical bows show that actions are performed simultaneously, show phrasing, and include body parts or add specific aspects to the movement.
Phrasing in movement" refers to how a sequence of movements is structured and connected, much like phrasing in music or language. It involves the flow and continuity of movements, highlighting the transitions and the relationship between different parts of the movement sequence. In the context of Laban Movement Analysis (LMA) and Labanotation, phrasing helps us to understand the dynamics, timing, and expressiveness of movement.

c) Basic form of the bow for "touch"—something is touching something.

5 At the far left the Effort signs are given connected with a travel line starting from the first sign next to the sign for the whole torso:

the "**Float**" Effort Action;
light, sustained, and indirect

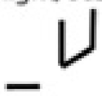

the "**Glide**" Effort Action;
light, direct, and sustained

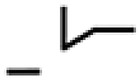

the "**Slash**" Effort Action;
strong, indirect, and quick

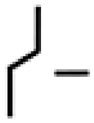

the "**Press**" Effort Action;
strong, direct, sustained

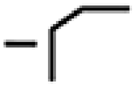

Finally, the movement is finished with the double horizontal lines, which always indicate that a movement is finished.

Wanda Ottes, June 2024.
